# Ablating putative Ku70 phosphorylation sites results in defective DNA damage repair and spontaneous induction of hepatocellular carcinoma

**DOI:** 10.1093/nar/gkab743

**Published:** 2021-08-24

**Authors:** Janapriya Saha, Jinsung Bae, Shih-Ya Wang, Huiming Lu, Lori J Chappell, Purva Gopal, Anthony J Davis

**Affiliations:** Division of Molecular Radiation Biology, Department of Radiation Oncology, UT Southwestern Medical Center, Dallas, TX, USA; Division of Molecular Radiation Biology, Department of Radiation Oncology, UT Southwestern Medical Center, Dallas, TX, USA; Division of Molecular Radiation Biology, Department of Radiation Oncology, UT Southwestern Medical Center, Dallas, TX, USA; Division of Molecular Radiation Biology, Department of Radiation Oncology, UT Southwestern Medical Center, Dallas, TX, USA; KBR, Houston, TX, USA; Department of Pathology, UT Southwestern Medical Center, Dallas, TX, USA; Division of Molecular Radiation Biology, Department of Radiation Oncology, UT Southwestern Medical Center, Dallas, TX, USA

## Abstract

Multiple pathways mediate the repair of DNA double-strand breaks (DSBs), with numerous mechanisms responsible for driving choice between the pathways. Previously, we reported that mutating five putative phosphorylation sites on the non-homologous end joining (NHEJ) factor, Ku70, results in sustained retention of human Ku70/80 at DSB ends and attenuation of DSB repair via homologous recombination (HR). In this study, we generated a knock-in mouse, in which the three conserved putative phosphorylation sites of Ku70 were mutated to alanine to ablate potential phosphorylation (Ku70^3A/3A^), in order to examine if disrupting DSB repair pathway choice by modulating Ku70/80 dynamics at DSB ends results in enhanced genomic instability and tumorigenesis. The Ku70^3A/3A^ mice developed spontaneous and have accelerated chemical-induced hepatocellular carcinoma (HCC) compared to wild-type (Ku70^+/+^) littermates. The HCC tumors from the Ku70^3A/3A^ mice have increased γH2AX and 8-oxo-G staining, suggesting decreased DNA repair. Spontaneous transformed cell lines from Ku70^3A/3A^ mice are more radiosensitive, have a significant decrease in DNA end resection, and are more sensitive to the DNA cross-linking agent mitomycin C compared to cells from Ku70^+/+^ littermates. Collectively, these findings demonstrate that mutating the putative Ku70 phosphorylation sites results in defective DNA damage repair and disruption of this process drives genomic instability and accelerated development of HCC.

## Graphical Abstract

**Graphical Abstract:**
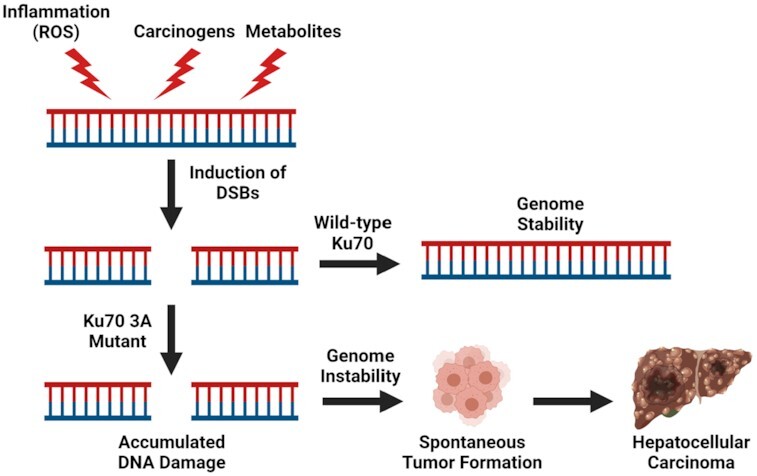
Mutating the putative Ku70 phosphorylation sites in a mouse model results in decreased DNA repair capacity, which induces chronic DNA damage and genomic instability and this subsequently drives the induction of spontaneous hepatocellular carcinoma. Graphical Abstract was created with BioRender.com.

## INTRODUCTION

The Ku protein, a heterodimer consisting of the subunits Ku70 and Ku80, plays critical roles in cellular processes such as non-homologous end joining (NHEJ), V(D)J recombination, apoptosis, telomere maintenance, aging, senescence and DNA replication (1–3). Ku is an abundant, highly conserved protein that possesses extremely high affinity for double-stranded (ds) DNA ends, sliding onto DNA via its ring-like structure that is generated by the intertwining of the subunits. It can bind various dsDNA ends, including those with blunt, overhangs and hairpins, and heterogeneous ends generated by ionizing radiation (IR). The most defined function of Ku is its role in the maintenance of genome integrity via initiation of the NHEJ pathway, the main DNA double strand break (DSB) repair pathway in mammalian cells (1,4). Following induction of a DSB, Ku70/80 binds to the DNA damage site within seconds after its creation and does so in all cell cycle phases (1,5,6). Ku recruits and activates the DNA-PK_cs_ kinase to the damage site, where the DNA-PK complex (DNA-PK_cs_-Ku-DNA) initiates the DNA damage response and chromatin remodeling (1,4,7). Once bound to the DSB, Ku performs its primary function in NHEJ, which is to serve as a scaffold to recruit the NHEJ machinery to the DNA lesion (4).

Delineating Ku's functions has been difficult in human cells because deletion of Ku70 is lethal and induced loss of Ku80 causes rapid cell death accompanied by massive telomere loss (8,9). However, Ku-deficient mouse models have been instrumental in examining its function in multiple pathways. Ku70^−/−^, Ku80^−/−^, Ku70^−/−^Ku80^−/−^ mice are viable and reproduce, but they show profound premature aging and are 40–60% the size of wild-type littermates (10–16). Consistent with the aging and growth defect phenotypes, mouse embryonic fibroblasts (MEFs) derived from Ku70^−/−^ and Ku80^−/−^ mice display premature senescence which is associated with an early loss of proliferating cells, a prolonged doubling time, and an accumulation of non-dividing cells (10,11,13). Ku80^−/−^ mice also exhibit premature age-specific changes including hepatocellular degeneration, hepatocellular inclusions, and hepatic hyperplastic foci (15). The role of Ku in protecting the genome is also supported by studies in various Ku-deficient rodent cell lines. Ku70- and Ku80-deficient MEFs and Chinese hamster ovary (CHO) cells are sensitive to various DNA damaging agents (11,13,15,17–19). V(D)J recombination requires NHEJ and not surprisingly, Ku-deficient mice are defective in this process, which results in substantial decrease in T- and B-cell production (10,11,13,14,16).

Although it is firmly established that Ku plays a pivotal role in maintaining genome integrity, its role as a tumor suppressor is not clearly defined. Ku70^−/−^ mice were found to have a significant incidence of thymic lymphomas (10,11). However, a subsequent study with Ku70^−/−^, Ku80^−/−^ and Ku70^−/−^Ku80^−/−^ mice with the same genetic background and raised in a similar environment found early aging in each cohort of mice, but without substantially increased cancer levels (12). Haplo-insufficiency of Ku80 in PARP^−/−^ mice (Ku80^+/–^PARP^−/−^ mice) promotes the development of hepatocellular adenoma and hepatocellular carcinoma (HCC), which correlated with elevated chromosomal aberrations (20). Ku70^−/−^ mice had accelerated HCC induction compared to wild-type and Ku70^+/–^ littermates after treatment with the liver carcinogen diethylnitrosamine (DEN) (21). The role of Ku70/80 in promoting tumor suppression in humans is mostly unknown, but a number of reports indicate that single nucleotide polymorphisms in Ku70 or Ku80 potentially contribute to different types of cancer including HCC, breast, lung, oral, bladder, renal and colorectal cancers (22).

A secondary function that Ku performs at DSBs is a general role of protecting DNA ends from non-specific processing, which if left unchecked could lead to genomic instability (1,6,23). Ku maintains the two ends of the broken DNA molecule together via forming a synaptic complex *in vitro* and is required for the positional stability of DSB ends *in vivo* (24,25). Ku binds to DSBs in all cell cycle phases including in S phase, the cell cycle stage where homologous recombination (HR) is the preferred DSB repair pathway (6,19). As DNA ends must be free for DNA end resection to occur in S phase, a mechanism must mediate the dissociation of the Ku heterodimer from DSBs in order to allow initiation of HR. In budding yeast, the Mre11–Rad50–Xrs2 (MRX) complex displaces yKu from DSB ends to drive pathway choice to HR (26). This is supported by studies that reported the human Mre11–Rad50–Nbs1 (MRN) complex removes Ku from DNA ends via an Mre11-dependent nucleolytic activity (27,28). It has also been reported that the E3 ubiquitin ligase RNF138 functions in the displacement of the Ku heterodimer from DSBs in order to promote HR (29). Previously, we identified that phosphorylation of Ku70 mediates the dissociation of the Ku heterodimer from DSBs to allow DNA end resection and the initiation of HR (19). This study drives our proposed model that phosphorylation-mediated dissociation of Ku from DSBs is one of the mechanisms that modulates DSB repair pathway choice in mammalian cells. However, the importance and physiological function of Ku70 phosphorylation *in vivo* is unknown. Using a mouse model with knock-in alanine substitutions (Ku70^3A/3A^ and Ku70^3A/+^) of the conserved putative Ku70 phosphorylation sites, we aimed to test our hypothesis that initiation of HR requires dissociation of Ku70/80 from DSB ends and that blocking this process results in enhanced genomic instability and tumorigenesis. In this study, we report that blocking Ku70 phosphorylation results in increased incidence of spontaneous and accelerated induction of chemical-induced HCC. Moreover, we found that Ku70^3A/3A^ mice and MEFs are radiosensitive and that Ku70^3A/3A^ MEFs have decreased DNA end resection and increased sensitivity to the DNA cross-linking agent mitomycin C (MMC), suggesting attenuation of HR. Collectively, the results suggest that mutating the putative Ku70 phosphorylation sites results in defective DNA repair and that this drives genomic instability and development of HCC.

## MATERIALS AND METHODS

### Cell culture

Primary MEFs isolated from E13.5 embryos from Ku70^+/+^ and Ku70^3A/3A^ mice were cultured in Hyclone α-MEM (GE Life Sciences) supplemented with 10% fetal bovine serum (Corning) and 1x penicillin/streptomycin (GE Life Sciences) and initially grown in an atmosphere of 3% O_2_ and 10% CO_2_ at 37°C in order to drive spontaneous transformation. Following transformation, the primary MEFs were grown in the same media in an atmosphere of 5% CO_2_. Ku70^−/−^ MEFs (11) were grown in Hyclone α-MEM supplemented with 5% fetal bovine serum, 5% newborn calf serum (GE Life Sciences), and 1x penicillin/streptomycin in an atmosphere of 5% CO_2_ at 37°C. Ku70^−/−^ MEFs complemented with mouse wild-type Ku70 and Ku70 3A were maintained in the same media but supplemented with 10 μg/ml hygromycin (Corning).

### Irradiation

Cells were irradiated with γ-rays generated by a Mark 1 ^137^Cs irradiator (J.L. Shepherd and Associates) at the doses denoted in the figures.

### Immunoblotting

Immunoblotting was performed as previously described (7). The following commercial antibodies were used: anti-tubulin (Sigma, T5168), anti-Ku70 (Santa Cruz, SC-17789) and anti-Ku80 (Santa Cruz, SC-515736). The secondary antibody used was the anti-mouse IgG (HRP-linked) from Cell Signaling Technology.

### Mouse Ku70 mutagenesis and generation of stable cell lines

Mouse Ku70 cDNA was PCR amplified using the primers mKu70-Bam-ATG: 5′-GGCGGATCCATGTCAGAGTGGGAGTCCTAC-3′ and mKu70-Stop-Xho: 5′-GGCCTCGAGTCAGTTCTTCTCCAAGTGTCTG-3′ and then subcloned into the pCDNA3.1-YFP-F2 mammalian expression vector using *Bam*HI and *Xho*I following standard protocols. PCR-directed mutagenesis using complimentary oligonucleotides was then used to generate mouse Ku70 3A. The following primers were used for the mutagenesis: mKu70-307A: 5′-GACTTTTAATGTAAACGCCGGCAGTCTACTCC-3′ and mKu70-314A/316A: 5′-CAGTCTACTCCTGCCTGCTGACGCCAAGCGGTCTCTGAC-3′ with the mKu70-307A primer set used first and then the mKu70-314A/316A primer set. To make Ku70^−/−^ MEFs that stably express wild-type and Ku70 3A, 4 μg of linearized plasmid containing wild-type or Ku70 3A cDNA was transfected using Amaxa nucleofector solution V (Lonza) and program T-020. Cells stably expressing mKu70 were selected using 800 μg/ml hygromycin.

### Live cell imaging and laser micro-irradiation

Live cell imaging combined with laser micro-irradiation was performed as previously described (6,7). Fluorescence was monitored via an Axiovert 200 M microscope (Carl Zeiss, Inc.), with a Plan-Apochromat 63×/NA 1.40 oil immersion objective (Carl Zeiss, Inc.). A 365-nm pulsed nitrogen laser (Spectra-Physics) was directly coupled to the epifluorescence path of the microscope and used to generate DSBs in a defined area of the nucleus. For quantitative analyses, the same amount of DNA damage was generated under standardized micro-irradiation conditions (minimal laser output of 75% for five pulses) in each experiment. Time-lapse images were taken with an AxioCamHRm camera. The fluorescence intensities of micro-irradiated and non-irradiated areas within the cell nucleus were determined using the AxioVision Software, version 4.8 (Carl Zeiss, Inc.). Each data point is the average of ≥10 independent measurements.

### RPA/Rad51 foci kinetics

IR-induced RPA/Rad51 kinetics were determined as previously outlined (30). RPA and Rad51 foci were detected using antibodies against RPA34 (NA-19L, EMD Millipore) or Rad51 (SC-8349, Santa Cruz), respectively. The images were acquired using a Zeiss Axio Imager fluorescence microscope utilizing a 63× oil objective. ≥100 cells were analyzed for each time point.

### 53BP1 foci kinetics

IR-induced 53BP1 kinetics were determined as previously outlined with modifications (19). Cells were grown on coverslips one day before the experiment and on the day of the experiment, cells were exposed to 1Gy of γ-rays. At different time points after IR (see figure), cells were washed twice with ice cold 1× PBS and fixed with 4% paraformaldehyde (in 1× PBS) for 20 min at RT, washed 5 times with 1× PBS, and incubated in 0.5% Triton X-100 on ice for 10 min. Cells were washed 5 times with 1× PBS and incubated in blocking solution (5% goat serum (Jackson Immuno Research) in 1× PBS) overnight. The blocking solution was then replaced with the 53BP1 (SC-22760, Santa Cruz) primary antibody diluted in 5% goat serum in 1x PBS and the cells were incubated for 2 h. Cells were then washed 5 times with wash buffer (1% BSA in 1× PBS). Cells were incubated with the Alexa Fluor 488 (1:1000) (Molecular Probes) secondary antibody in 1% BSA, 2.5% goat serum in 1× PBS for 1 h in the dark, followed by five washes. After the last wash, cells were mounted in VectaShield mounting medium containing DAPI. The images were acquired using a Zeiss Axio Imager fluorescence microscope utilizing a 63× oil objective. ≥100 cells were analyzed for each time point.

### Mice

All mice were approved by and handled in accordance with the guidelines of the Institutional Animal Care and Use Committee (IACUC) at UT Southwestern Medical center (UTSW). All mice were bred and maintained in a specific pathogen free SPF barrier vivarium at UTSW. All experiments were conducted with age and sex matched mice and treatment groups were allocated randomly.

### Generation of Ku70^3A/3A^ knock-in mice

ES cell targeting and generation of chimeric mice were performed at the transgenic core facility at UTSW. Specifically, C57BL/6N females (Charles River) were induced to superovulate by injecting pregnant mare serum gonadotropin (PMSG) and human chorionic gonadotropin (HCG) and then mated with C57BL/6N males to collect the zygotes for microinjection. A mixtures of Cas9-mRNA (Sigma) (50 ng/μl), synthesized gRNA (Sigma) (25 ng/μl) and template single-strand oligodeoxynucleotide (ssODN) (Sigma) (25 ng/μl) were injected into pronuclear of one-cell zygotes. The Ku70 gRNA sequence utilized is 5′-ACACCAAGCGGTCTCTGGTAGG-3′ and the template ssODN (donor oligo) sequence is 5′-GAACCAGTGAAAACCAAGACAAGGACTTTTAATGTAAACGCCGGCAGTCTACTCCTGCCTGCTGACGCCAAGCGGTCTCTGGTAGGTGGCTAACCTTTCCTACCGAATCTTGTTTAAGA-3′ (the mutation sites are underlined). After microinjection, the zygotes were implanted into ICR recipients, which carried them to term. Seven chimeras (7 out of 24 total pups, 29.2% efficiency) were obtained and then backcrossed twice with wild-type C57BL/6J mice (Jackson Laboratory). For sequencing and PCR genotyping, DNA was isolated from either the mouse tail or ear using a previously described protocol (31). For sequencing, the following primers were used to amplify Ku70 sequencing, mKu70-1129F (5′-TTGGACAGAGTAACGCAGACTGG-3′) and mKu70-11802R (5′-CTCGTGCATGCCTCATGCATGC-3′). Sequencing was performed at the UTSW Sanger Sequencing Core. For genotyping of mice via PCR analysis, the following primer sets were used: Wild-type- primers mKu70-WT (5′-CGGCAGTCTACTCCTGCCTAG-3′) and mKu70-11802R and Ku70-3A- primers mKu70-314A (5′- CGGCAGTCTACTCCTGCCTGC-3′).

### Monitoring of mice and examination of tumorigenesis

Ku70^3A/3A^, Ku70^3A/+^ and Ku70^+/+^ littermates were maintained and monitored for up to 28 months. Necropsy was performed at various time points and the mice were examined for gross tumors. All tissues with tumors were collected and fixed with 4% neutralized PFA for 24 hours. Clinical histopathological analysis of H&E-stained sections were performed blindly.

### Diethylnitrosamine (DEN)-induced HCC model

Male C57BL/6J Ku70^3A/3A^ and Ku70^+/+^ littermates were injected with DEN (Sigma) (25 mg/kg i.p.) intraperitoneally (i.p.) at day 14 postpartum. All mice were monitored weekly for weight and activity and sacked at different intervals (4, 6, 9 months) as appropriate to test for liver disease. Nine months following DEN injection, all surviving mice from all genotypes were euthanized with CO_2_ and their liver tissue was collected and fixed. DEN-induced liver tumor numbers were determined visually, and size determined using a Vernier caliper.

### Immunohistochemistry (IHC)

The fixed tissues samples were processed for paraffin sectioning and stained with hematoxylin-eosin (H&E) according to standard protocol by the Histo-Pathology Core and Tissue Management Shared Resource (TMSR) at UTSW. IHC staining was performed using the following antibodies, Phospho-Histone H2A.X (Ser139) (20E3) Rabbit mAb (Cell Signaling), Ki-67 (D3B5) Rabbit mAb (Cell Signaling), and DNA/RNA damage antibody (8-oxo-G) [15A3] (Abcam) by the TMSR at UTSW.

### Total body irradiation survival experiments

Healthy 8-week-old Ku70^3A/3A^ mice and Ku70^+/+^ littermates were irradiated with 9 Gy total body irradiation (TBI) utilizing the self-contained X-ray irradiation system (XRAD 320, Precision X-Ray). Their weight and activity were monitored daily until 30 days post-irradiation. The mice were sacked when they lost more than 20% of their initial body weight; their Body Conditioning Score (BCS) fell below 2%, or at the date of experimental termination.

### Clonogenic survival assays

For clonogenic survival assays, DC-1 (Ku70 null) and DC-1 complemented with wild-type mouse Ku70 or Ku70 3A protein or spontaneously transformed MEFs from Ku70^+/+^ or Ku70^3A/3A^ (clones #51 and #57) were treated with increasing doses of DNA damaging agents (γ-rays or MMC, see figures for doses) and survival assay were performed as previously outlined (7,19). Furthermore, MEFs from Ku70^+/+^ or Ku70^3A/3A^ (clones #51 and #57) were pre-treated with 1 μM of the DNA-PK_cs_ inhibitor NU7441 (Selleck Chemicals) for 2 h before irradiation in the experiment shown in Figure 6E.

### Chromosomal abnormalities

For chromosomal abnormalities, spontaneously transformed primary MEFs (passages 8–10) were incubated with 200 ng/ml MMC (Sigma) for 20 h followed by addition of 0.1 μg/ml Demecolcine Solution (Sigma) in the last 4 h. Samples were processed and chromosomal abnormalities were scored as previously described (32).

### Isolation of splenic immune cells

Total immune cells from the spleen were isolated following previously reported methods (33).

### Staining of splenic immune cells

For cell surface staining, lymphocytes isolated from spleens were mixed with anti-mouse CD16/32 mAbs on ice for 5 min to block Fc receptors. Cells were mixed with Ab cocktail containing mAbs against specific cell-surface markers and incubated on ice for 30 min in the dark. The following antibodies were used (all purchased from eBioscience (San Diego, CA, USA, now Affymetrix, part of Thermo Fisher Scientific), Panel 1 (Ly6G-FITC, F4/80-PE, CD11b-PE-Cy7, Ly6C-PerCP-Cy5.5 and CD11c-APC) and Panel 2 (CD19-FITC, CD25-PE, CD3-PE-Cy7, CD8-PerCP-Cy5.5, CD4-AF700 and CD49b-eFluor450). The stained samples were transferred to 5-ml flow tubes and 200 μl of staining buffer was added to each sample. The samples were run on a FACS Calibur (BD) and data analyzed using FlowJo software by the Flow Cytometry Core at UTSW.

## RESULTS

### Three of five putative Ku70 phosphorylation sites are found in mice and mutating these sites to alanine attenuates DNA end resection and onset of homologous recombination

**Figure 1.**
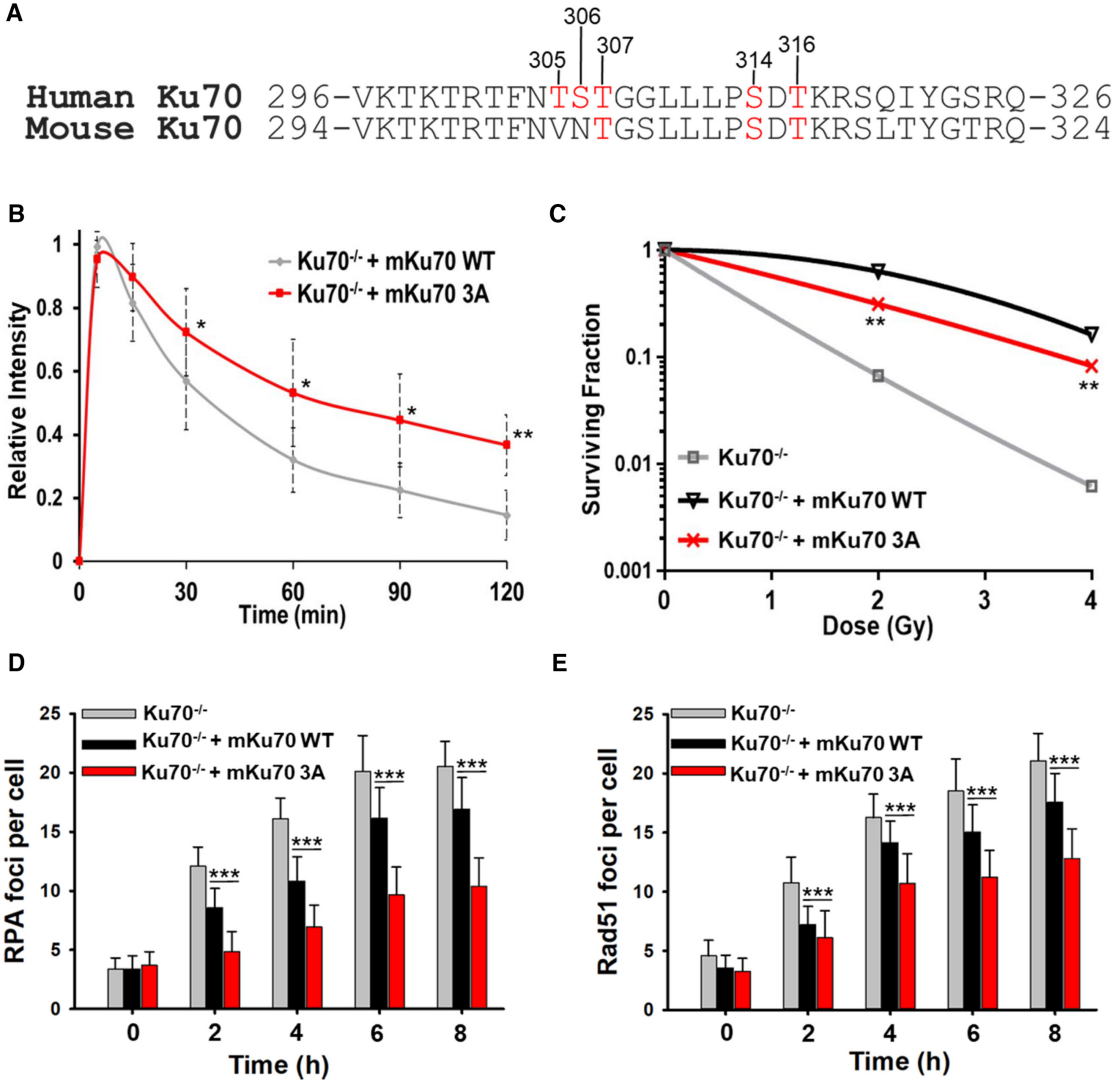
Three of the five putative Ku70 phosphorylation sites are conserved in mice and mutating these sites to alanine attenuates DNA end resection and onset of homologous recombination. (**A**) Alignment of human and mouse Ku70 amino acid sequences. Putative phosphorylation sites are in red. (**B**) Relative fluorescence intensity of YFP-tagged Ku70 and Ku70 3A proteins in Ku70^−/−^ MEFs (DC-1) at DSBs after micro-irradiation. (**C**) Clonogenic survival assays were performed to compare the radiation sensitivities of Ku70^−/−^ MEFs or Ku70^−/−^ MEFs complemented with mouse Ku70 wild-type (mKu70 WT) or 3A (mKu70 3A). Cells were irradiated at the indicated doses and plated for analysis of survival and colony-forming ability. Error bars denote the standard deviation values. (D and E) Immunostaining for RPA (**D**) or Rad51 (**E**) in Ku70^−/−^ MEFs or Ku70^−/−^ MEFs complemented with Ku70 wild-type (mKu70 WT) or 3A (mKu70 3A) after exposure to 8 Gy. Cells were pre-extracted and fixed 2, 4, 6 or 8 h after IR and immunostained for RPA or Rad51. RPA and Rad51 foci were counted in EdU positive cells (S phase) and averaged. Data from at least three separate experiments (n ≥ 3) are displayed ± standard error of the mean (SEM) unless specified otherwise. Student's t-test (two-sided) was performed to assess statistical significance (*P < 0.05, **P < 0.01, ***P < 0.001).

We previously reported that phosphorylation at a cluster of sites in human Ku70 mediates the dissociation of the Ku heterodimer from DSBs and that blocking phosphorylation of these sites attenuates HR (19). An alignment of DNA sequences of Ku70 orthologues found that the five sites are conserved only in primates, whereas T307, S314 and T316 are conserved in vertebrates, including in mice (Figure 1A and Supplementary Figure S1). To test the functionality of the conserved sites in mice, they were mutated in mouse Ku70 cDNA to alanine to ablate potential phosphorylation (Supplementary Figure S2A) and then stably expressed in the mouse Ku70^−/−^ cell line DC-1 (Supplementary Figure S2B). Similar to our previous complementation studies with the human Ku70 5A mutant (19), the mouse Ku70 3A protein is retained at micro-irradiation generated DSBs (Figure 1B and Supplementary Figure S2C and D) and caused moderate radiosensitivity compared to DC-1 cells complemented with wild-type mouse Ku70 (Figure 1C). Furthermore, cells expressing mouse Ku70 3A showed decreased DNA end resection and ongoing HR, as monitored by IR-induced RPA and Rad51 foci resolution, respectively (Figure 1D and E, Supplementary Figure S3A and B). Collectively, the data show that three of the five putative Ku70 phosphorylation sites are conserved in mice and that mutating these sites to alanine in mouse Ku70 results in similar phenotypes as observed with human Ku70 mutants (19).

### Generation of Ku70^3A/3A^ mice

**Figure 2.**
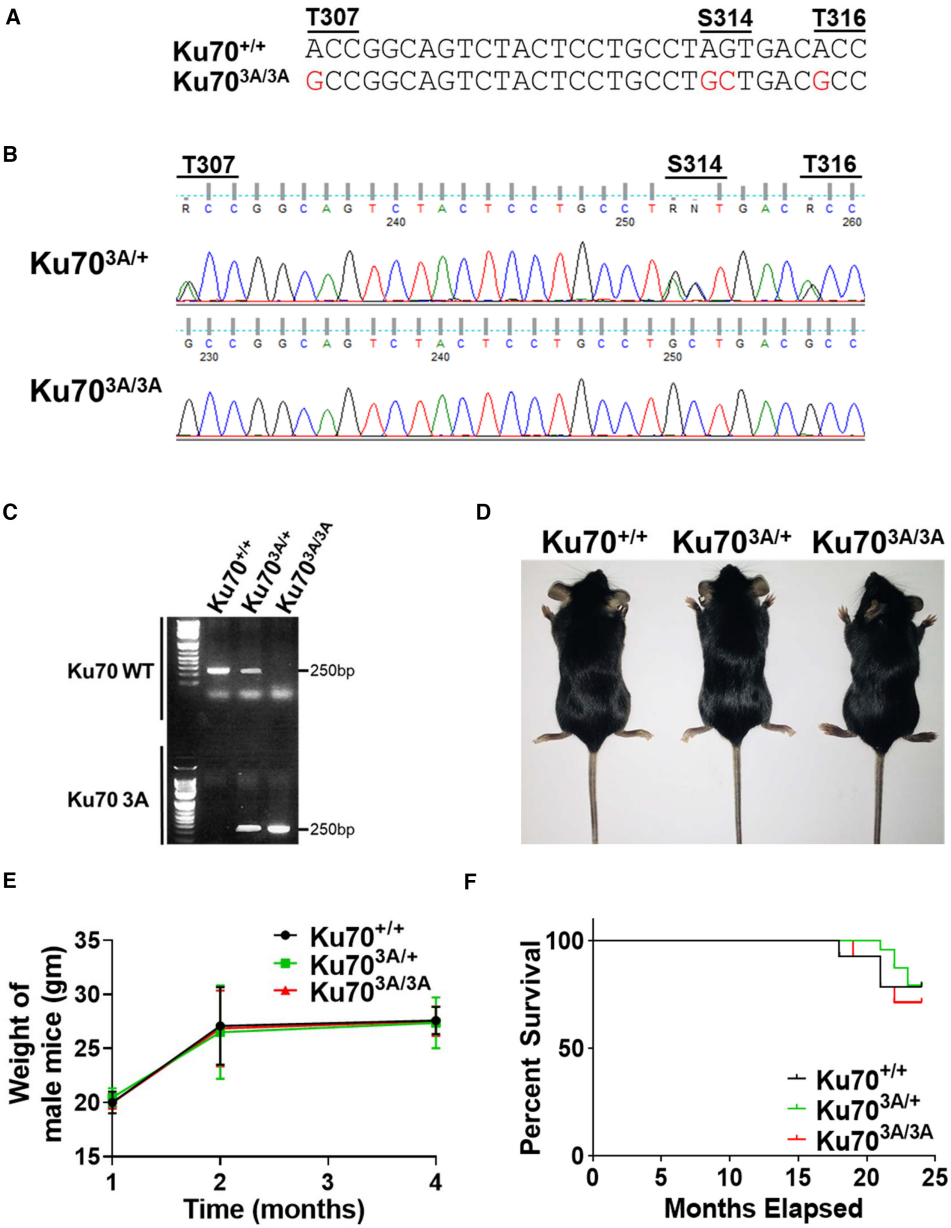
Generation of Ku70^3A/3A^ and Ku70^3A/+^ mice and initial characterization. (**A**) Nucleotides changed to ablate phosphorylation of putative mouse Ku70 phosphorylation sites. Sequencing (**B**) and PCR (**C**) verification of Ku70^3A/3A^ and Ku70^3A/+^ mouse genotypes. (**D**) Image of Ku70^+/+^, Ku70^3A/+^, Ku70^3A/3A^ mice. (**E**). Growth of male Ku70^+/+^, Ku70^3A/+^, Ku70^3A/3A^ mice was tracked by weight. Error bars denote SD values for the weight of five mice of each genotype. (**F**). Kaplan-Meier survival curves for Ku70^+/+^ (*n* = 14), Ku70^3A/+^ (*n* = 24), Ku70^3A/3A^ (*n* = 14) mice for 24 months.

To examine the impact of mutating the putative Ku70 phosphorylation sites *in vivo*, we generated a C57BL/6J mouse model with alanine substitutions in the three conserved putative phosphorylation sites (Ku70^3A/3A^) using CRISPR/Cas9 (Figure 2A–D). Seven chimeras were obtained and then backcrossed twice with wild-type C57BL/6J mice in order to limit potential off-target mutations generated by CRISPR/Cas9. The resulting Ku70^3A/+^ mice were used and intercrosses between Ku70^3A/+^ mice produced Ku70^3A/3A^ mice at the expected Mendelian ratio. Furthermore, the Ku70^3A/3A^ male and female mice were fertile. Sequencing and PCR analysis validated the Ku70^3A/+^ and Ku70^3A/3A^ genotypes and further studies were performed using three separate founder lines (Figure 2B and C). Ku70^+/+^, Ku70^3A/+^, Ku70^3A/3A^ mice were tracked for growth once a month until 4 months of age and then every 2 months until the mice were 24 + months old. All three genotypes (males and females) developed normally (Figure 2D and E and Supplementary Figure S4), and life span analysis showed that there was no significant difference between the three groups of mice up to 24 months of age (Figure 2F). Since Ku70^−/−^ mice lack mature B cells and serum immunoglobulin and develop only small populations of thymic cells, we analyzed Ku70^3A/3A^ mice for lymphocyte development (10–12,18). The spleens of 8-week-old Ku70^+/+^ and Ku70^3A/3A^ mice were isolated and fluorescence activated cell sorting analyses of immune cells were performed. The immune phenotyping analyses of splenic immune cells did not reveal any significant differences in the lymphoid (T- and B-cell populations), T-cell sub-populations (CD4 and CD8 cells; CD4 sub-population CD25 – regulatory T cells) and Natural Killer (NK) cells or myeloid cell populations (neutrophils, macrophages, monocytes and dendritic cells) between Ku70^3A/3A^ and Ku70^+/+^ littermates (Supplementary Figure S5). As Ku70^3A/3A^ mice develop normally, this illustrates that these mice are distinct phenotypically from Ku70-deficient mice, which have severe growth retardation, premature aging, and defective V(D)J recombination (10–12,18).

### Ku70^3A/3A^ and Ku70^3A/+^ mice develop spontaneous hepatocellular carcinoma (HCC)

**Figure 3.**
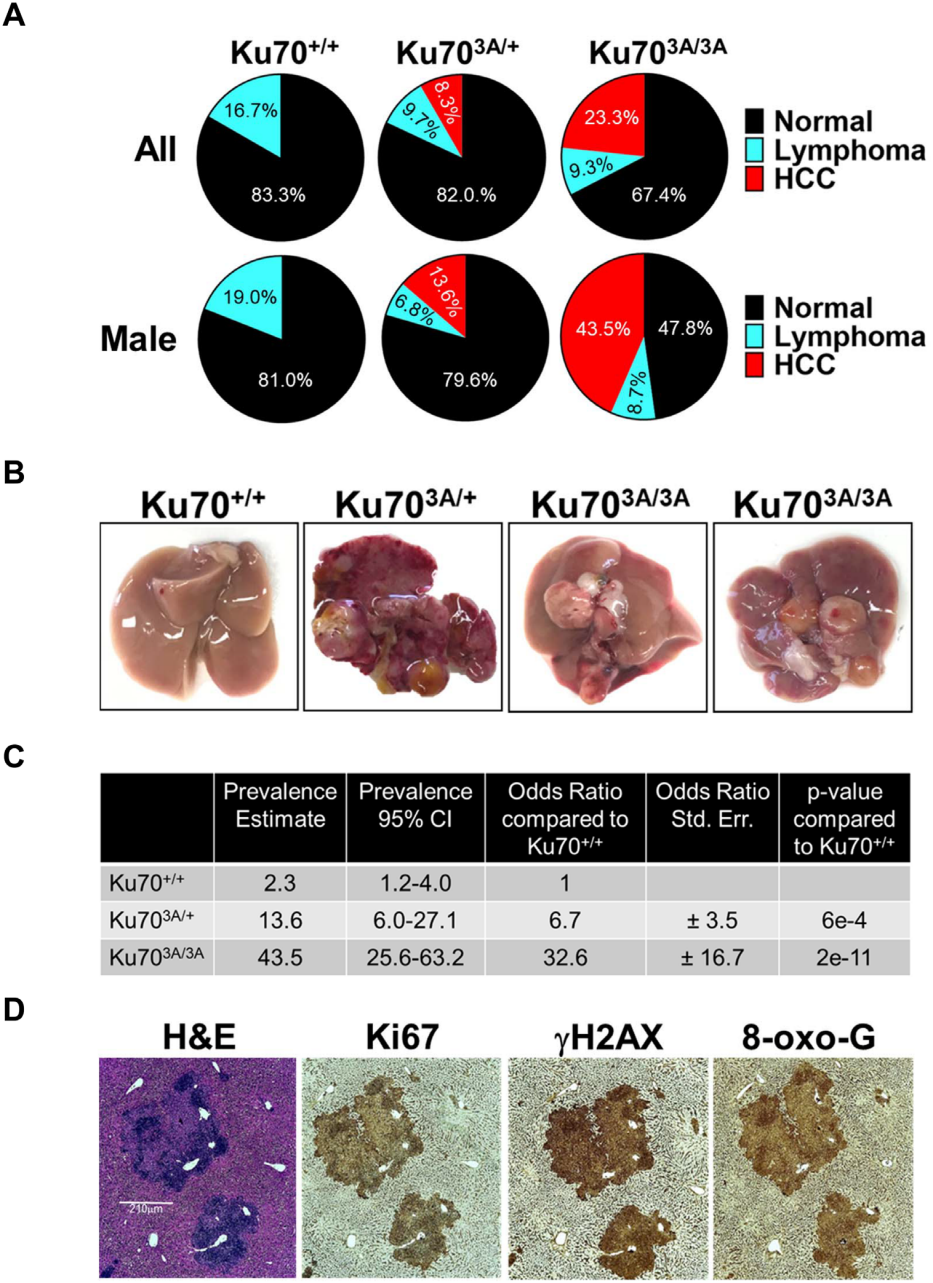
Ku70^3A/3A^ and Ku70^3A/+^ mice have increased incidence of spontaneous hepatocellular carcinoma (HCC). (**A**) Tumor incidence in all (upper panel) and male (lower panel) Ku70^+/+^, Ku70^3A/+^ and Ku70^3A/3A^ mice. Number of mice analyzed were 30 overall and 21 males for Ku70^+/+^, 72 overall and 44 males for Ku70^3A/+^, and 42 overall and 22 males for Ku70^3A/3A^. (**B**) Representative images of livers and HCCs from Ku70^+/+^, Ku70^3A/+^ and Ku70^3A/3A^ mice. (**C**) HCC tumor prevalence in Ku70^+/+^, Ku70^3A/+^ and Ku70^3A/3A^ mice with 95% confidence interval (CI) and HCC tumor odds ratio (OR) for Ku70^3A/+^ and Ku70^3A/3A^ compared to Ku70^+/+^ mice. Standard errors and p-values are also provided. (**D**) Immunohistochemistry staining of HCC tumor from a selected Ku70^3A/3A^ mouse. IHC staining was performed using hematoxylin and eosin (H&E) stain and the following antibodies, phospho-Histone H2A.X (Ser139) (γH2AX), Ki67, and 8-oxoguanine (8-oxo-G).

No differences in survival rate were observed in our cohort of mice (Figure 2F), but necropsy and clinical histopathological analysis surprisingly revealed development of HCC in the Ku70^3A/3A^ and Ku70^3A/+^ mice (Figure 3). No HCC was identified in Ku70^+/+^ mice; however, HCC was detected in 8.3% of Ku70^3A/+^ and 23.3% of Ku70^3A/3A^ mice, including HCC being observed as early as 17 months in Ku70^3A/3A^ mice (Figure 3A). Similar to previous mouse studies, development of HCC occurred more often in male than female mice (34). Spontaneous HCC was only identified in male mice in the Ku70^3A/3A^ cohort with 43.5% (95% CI for HCC tumor prevalence was 25.6–63.2%) of male mice developing HCC (Figure 3A–C). One female in the Ku70^3A/+^ cohort developed HCC and the incidence of male Ku70^3A/+^ developing HCC was 23.3% (95% CI for HCC tumor prevalence was 6.0–27.1%) (Figure 3A–C). We performed logistic regression analysis to estimate an odds ratio comparing Ku70^+/+^ to both Ku70^3A/+^ and Ku70^3A/3A^ male mice. As none of our Ku70^+/+^ male mice developed HCC, we used historical data of spontaneous HCC induction in untreated mice in combination with our own data for the logistic regression analysis (35,36). The analysis found that the odds of HCC tumorigenesis is 32.63 times greater (95% CI is 11.96–89.02) in Ku70^3A/3A^ mice and 6.7 times greater (95% CI 2.38–18.83) in Ku70^3A/+^ mice compared to Ku70^+/+^ mice (Figure 3C). To further characterize the effects of mutating the putative Ku70 phosphorylation sites on HCC development, we examined liver sections of Ku70^3A/3A^ mice with pathology verified HCC by immunohistochemistry (IHC) staining with the proliferation marker Ki67, DSB marker γH2AX, and free radical damage/reactive oxygen species (ROS) marker 8-oxoguanine (8-oxo-G). IHC analysis of the HCC tumors from Ku70^3A/3A^ revealed strong Ki67, γH2AX and 8-oxo-G staining, suggesting high levels of proliferation and accumulated DNA damage in the HCC tumors (Figure 3D). Pathologic analysis also revealed spontaneous lymphoma in all three groups of mice (Figure 3A), which is not surprising as C57BL/6J mice are prone to lymphomas and hematopoietic neoplasia (35,37). However, no increase in lymphoma was observed in the Ku70^3A/+^ and Ku70^3A/3A^ compared to the Ku70^+/+^ mice. Moreover, no lymphomas were found in any mouse that was less than 24 months old, suggesting this phenomenon is due to the propensity of aged C57BL/6J to develop lymphoma. Collectively, the data Ku70^3A/3A^ and Ku70^3A/+^ mice develop spontaneous HCC and this correlates with accumulated DNA damage.

### Ku70^3A/3A^ mice have accelerated chemical-induced HCC development

**Figure 4.**
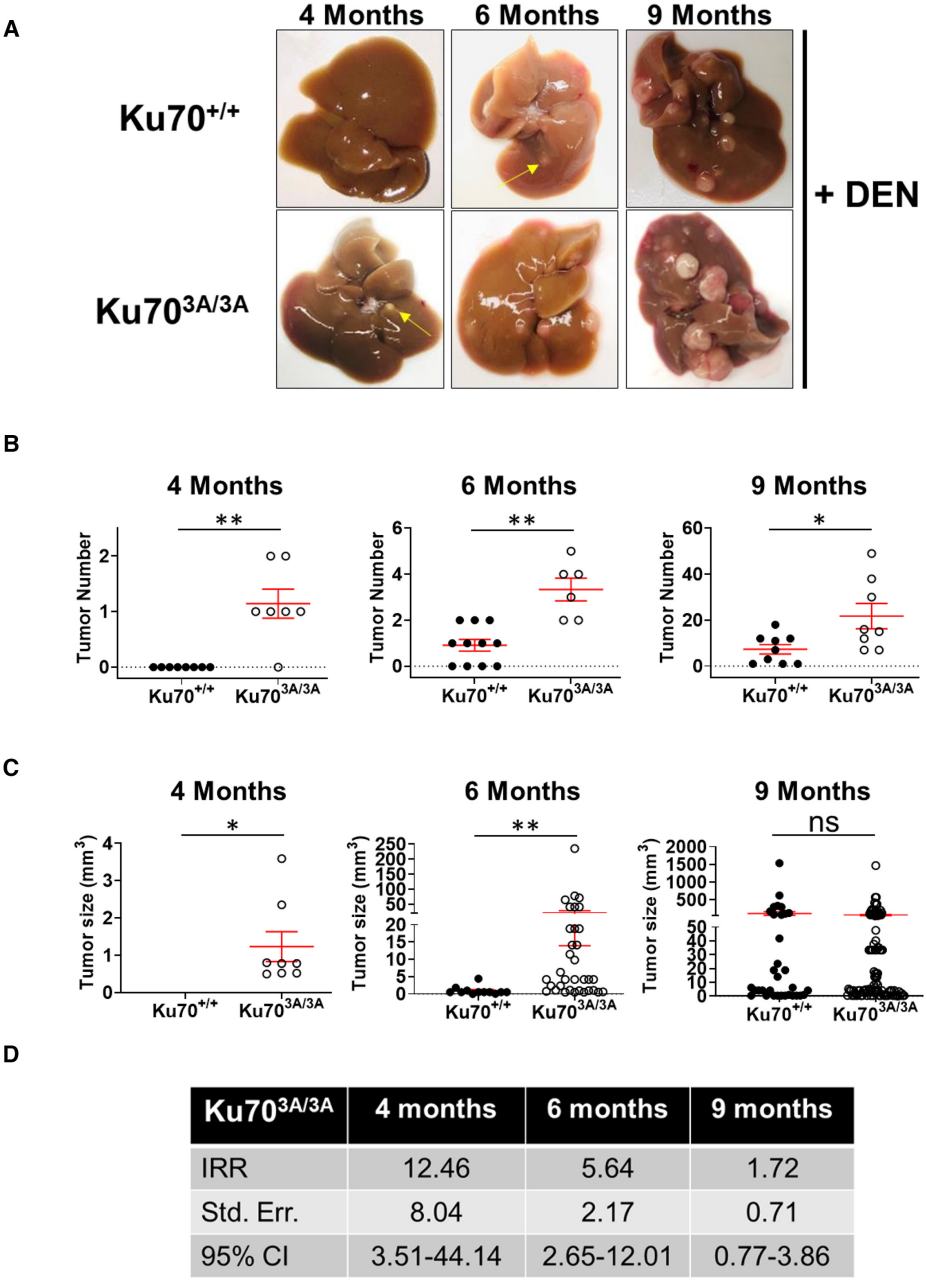
Ku70^3A/3A^ mice have accelerated diethylnitrosamine (DEN)-induced HCC compared to Ku70^+/+^ littermates. (**A**) Representative images of livers and HCCs from Ku70^+/+^ and Ku70^3A/3A^ mice 4, 6 and 9 months following injection of the liver carcinogen, DEN. DEN-induced tumor number (**B**) and tumor size (**C**) in Ku70^+/+^ and Ku70^3A/3A^ mice 4, 6 and 9 months following DEN injection. SEM is shown and Welch's t-test was used to examine statistical significance. (*P < 0.05, **P < 0.01, and ns = no significance). (**D**) Negative binomial regression was used to calculate incidence rate ratio (IRR) for DEN-induced tumorigenesis. Standard error and 95% confidence interval (CI) are also provided.

Since spontaneous HCC development in the Ku70^3A/3A^ mice correlates with accumulated DNA damage and thus defective DNA repair, we next examined if induction of DNA damage via the liver carcinogen diethylnitrosamine (DEN) promotes accelerated HCC development in Ku70^3A/3A^ mice (38). DEN induces acute liver injury by damaging DNA by directly generating DNA adducts and inter-strand crosslinks (ICLs) and indirectly via ROS production during metabolism of the drug that generates single strand breaks (SSBs) and DSBs (39,40). DEN works in a dose-dependent manner and DEN-induced HCC development is faster in young mice and males (39). In this study, 14-day-old Ku70^+/+^ and Ku70^3A/3A^ mice were treated with a single dose (25 mg/kg, intraperitoneally) of DEN and were examined at 4-, 6-, and 9-months post-injection for liver tumors. A minimum of 6 (and up to 11) animals from each genotype were analyzed. There was no evidence of liver neoplasia in the Ku70^+/+^ mice at 4 months, whereas 6 out of 7 Ku70^3A/3A^ mice had at least 1 liver tumor at this time point (average of 1.14 ± 0.26 tumors/mouse) (Figure 4A and B). Furthermore, at 6 months, all (6 out of 6) Ku70^3A/3A^ mice showed liver tumors, with an average of 3.33 ± 0.49 tumors/mouse (Figure 4A and B). At 6 months, 7 out of 11 Ku70^+/+^ mice had liver tumors, but the average was only 0.91 ± 0.25 tumors/mouse. Consistent with an earlier induction of HCC in the mutant mice, tumors found in 6-month-old Ku70^3A/3A^ mice were significantly larger (21.42 ± 7.53 mm^3^) than those found in Ku70^+/+^ mice (0.91 ± 0.34 mm^3^) (Figure 4C). Expectedly, at 9 months, all mice in each genotype cohort had DEN-induced HCC, but the Ku70^3A/3A^ mice had significantly higher tumor numbers (21.75 ± 5.48 tumors/mouse) compared to Ku70^+/+^ mice (7.33 ± 2.07) with no difference in average tumor size between the two cohorts observed (Figure 4A−C). Negative binomial regression analysis was utilized to calculate incidence-rate ratio for tumor development at each time point. The incidence rate of DEN-induced tumor formation for Ku70^3A/3A^ mice is 12.5 times (95% CI 3.51–44.14) higher at 4 months, 5.6 times (95% CI 2.65–12.01) higher at 6 months, and 1.7 (95% CI 0.77–3.86) times higher at 9 months compared to Ku70^+/+^ mice (Figure 4D). Control (DEN-untreated) Ku70^3A/3A^ and Ku70^+/+^ mice did not develop any tumors when the experiment was terminated 9 months post-treatment. Collectively, Ku70^3A/3A^ mice develop spontaneous and have accelerated chemical-induced HCC compared to Ku70^+/+^ littermates.

### Ku70^3A/3A^ mice are radiosensitive

**Figure 5.**
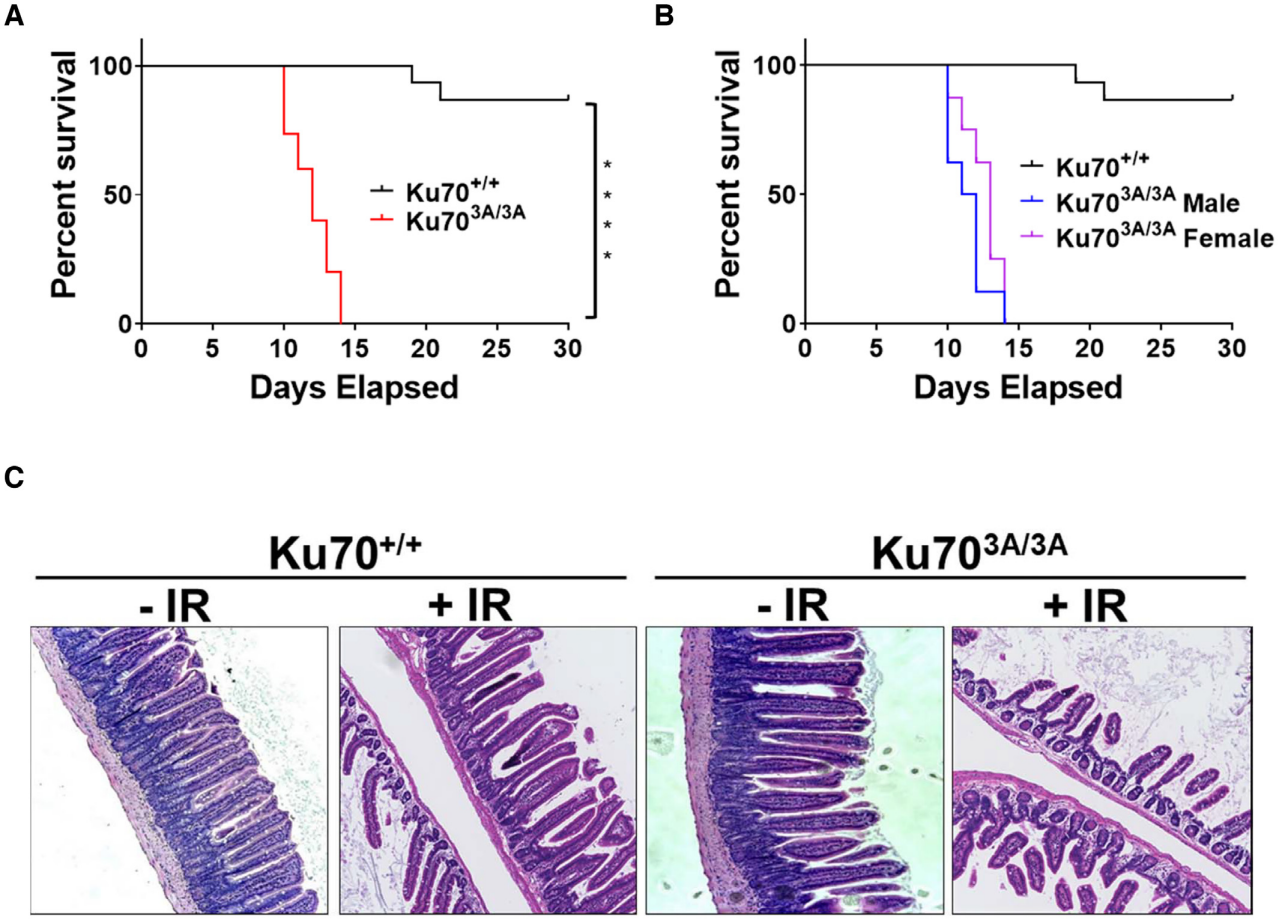
Ku70^3A/3A^ mice are radiosensitive. Kaplan-Meier survival curves for all (**A**) and male vs. female (**B**) Ku70^+/+^ and Ku70^3A/3A^ mice following total body irradiation with 9 Gy. Number of mice analyzed were 15 for Ku70^+/+^ and 16 (8 males and 8 females) for Ku70^3A/3A^. Log rank test was used to examine statistical significance (****P < 0.0001). (**C**). Hematoxlin and eosin (H&E) stain of colon of Ku70^+/+^ and Ku70^3A/3A^ mice 10 days following total body irradiation.

The liver tumors in Ku70^3A/3A^ mice have accumulated DNA damage, suggesting defective DNA repair in this cohort of mice. To test if the Ku70^3A/3A^ mice have defective DSB repair compared to their Ku70^+/+^ littermates, total body irradiation (TBI) experiments were performed. Treatment with 9 Gy of X-rays revealed that Ku70^3A/3A^ mice are significantly radiosensitive compared to Ku70^+/+^ mice (Figure 5A). All (15 out of 15) Ku70^3A/3A^ mice succumbed to radiation injury between days 10 and 14, whereas by day 30, only 13.3% (2 out of 15) of Ku70^+/+^ mice had perished from the radiation exposure. No statistical difference between male and female Ku70^3A/3A^ mice in radioresponse was observed (Figure 5B). The radiosensitivity of the Ku70^3A/3A^ mice correlated with classic acute radiation syndrome (ARS) symptoms, as intestinal H&E staining showed significant intestinal crypt and villi destruction in the Ku70^3A/3A^ mice compared to Ku70^+/+^ (Figure 5C).

### Mouse embryonic fibroblasts (MEFs) isolated from Ku70^3A/3A^ mice are defective in DNA repair

**Figure 6.**
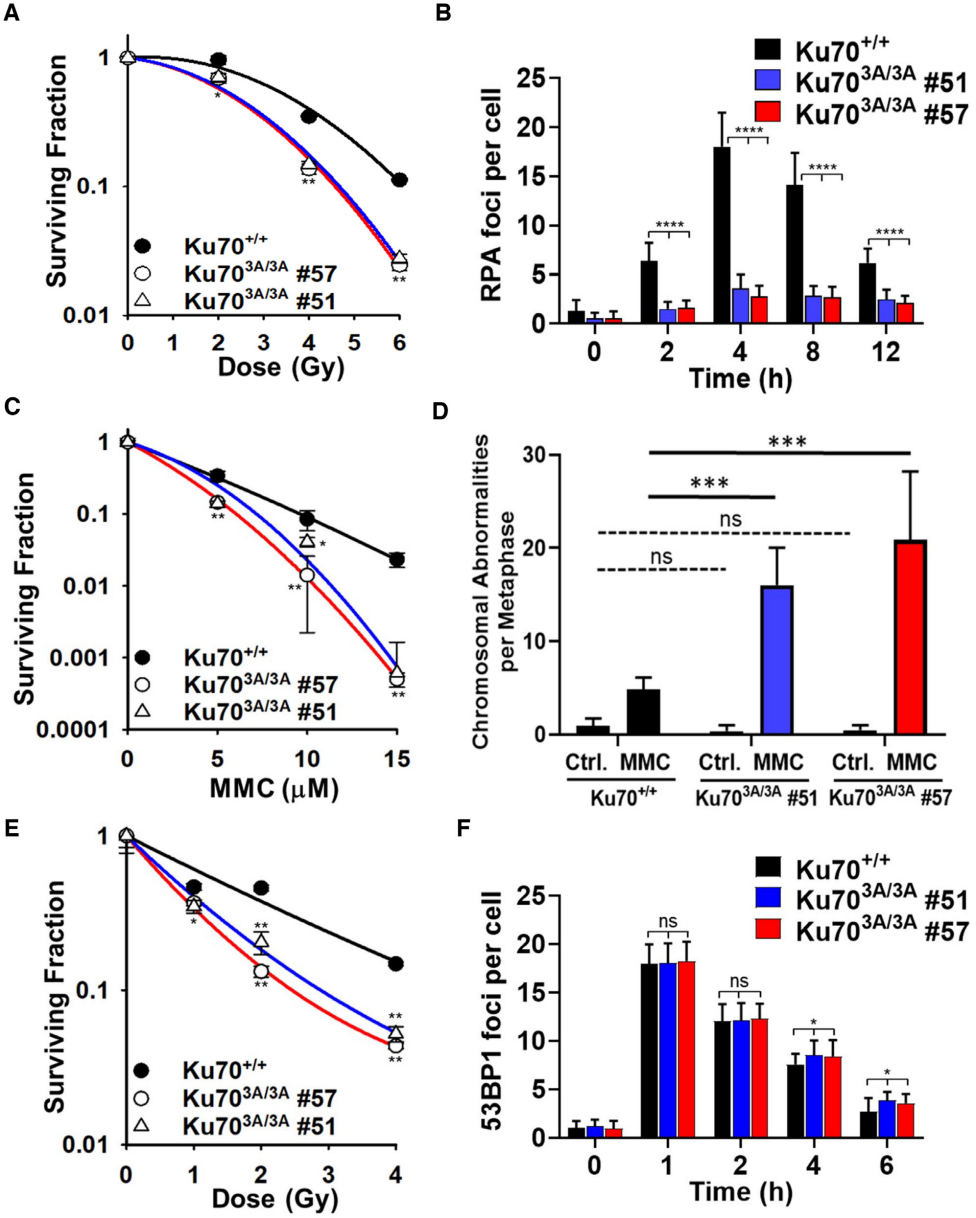
Mouse embryonic fibroblasts (MEFs) isolated from Ku70^3A/3A^ mice are radiosensitive, have attenuated IR-induced DNA end resection, and are sensitive to the DNA cross-linking agent mitomycin C (MMC). (**A**) Clonogenic survival assays were performed to compare the radiation sensitivities of MEFs isolated from a Ku70^+/+^ mouse and two separate Ku70^3A/3A^ mice (clones #51 and #57). Cells were irradiated at the indicated doses and plated for analysis of survival and colony-forming ability. (**B**) Immunostaining of RPA foci in Ku70^+/+^ and Ku70^3A/3A^ MEFs after treatment with 8 Gy. Cells were pre-extracted and fixed 2, 4, 8 or 12 h after IR and immunostained for RPA. RPA foci in EdU positive cells (S phase) were counted for each cell and averaged. Error bars denote standard error of the mean (SEM). (**C**) Clonogenic survival assays were performed to compare the sensitivities of Ku70^+/+^ and Ku70^3A/3A^ MEFs to MMC. Cells were treated with MMC at the indicated doses and plated for analysis of survival and colony-forming ability. (**D**) Metaphase spreads were performed following treatment with MMC in Ku70^+/+^ and Ku70^3A/3A^ MEFs and chromosomal abnormalities were enumerated. Error bars denote SEM values and Welch's t-test was performed to assess statistical significance (ns = no significance, ***P < 0.001). (**E**) Clonogenic survival assays were performed to compare the radiation sensitivities of Ku70^+/+^ and Ku70^3A/3A^ MEFs in the presence of the DNA-PK_cs_ inhibitor NU7441 (1 μM). Cells were pretreated with NU7441 for 2 hours and then irradiated at the indicated doses and plated for analysis of survival and colony-forming ability. (**F**) Immunostaining of 53BP1 foci in Ku70^+/+^ and Ku70^3A/3A^ MEFs after exposure to 1 Gy. Cells were fixed 1, 2, 4 or 6 h after IR and immunostained for 53BP1 foci. 53BP1 foci were counted for each cell and averaged. Data from at least three separate experiments (*n* ≥ 3) are displayed ± standard deviation unless specified otherwise. Student's *t*-test (two-sided) was performed to assess statistical significance (*P < 0.05, **P < 0.01, ***P < 0.001, ****P < 0.0001, and ns = no significance) unless otherwise noted.

Ku70^3A/3A^ mice are radiosensitive, suggesting defective DNA repair. To further examine DNA repair processes in this setting, we isolated MEFs from a Ku70^+/+^ mouse and two Ku70^3A/3A^ littermates (Clones #51 and #57) and immunoblotting showed similar protein expression of Ku70 and Ku80 in these MEFs (Supplementary Figure S6A). The two Ku70^3A/3A^ MEFs were radiosensitive compared to those obtained from Ku70^+/+^ littermates (Figure 6A). Ku70^3A/3A^ MEFs also showed decreased IR-induced RPA foci formation compared to Ku70^+/+^ MEFs, illustrating that mutating the putative Ku70 phosphorylation sites in the mouse cells results in a significant decrease in DNA end resection and likely a decrease in HR (Figure 6B and. Supplementary Figure S6B). Insufficient transfection rates in the Ku70^3A/3A^ MEFs resulted in the inability to examine HR rates using a HR GFP reporter assay; therefore, we compared the sensitivity of Ku70^3A/3A^ and Ku70^+/+^ MEFs to the DNA cross-linking agent, mitomycin C (MMC). MMC-induced ICLs are effectively resolved/repaired via the Fanconi anemia (FA) pathway in conjunction with HR (41). Thus, an increase in MMC sensitivity can be used as an indirect test for HR-deficiency. As shown in Figure 6C, the two Ku70^3A/3A^ MEF cell lines are more sensitive to MMC than Ku70^+/+^ MEFs. Furthermore, the Ku70^3A/3A^ MEFs showed significantly higher MMC-induced chromosomal abnormalities compared to their Ku70^+/+^ counterparts (Figure 6D and Supplementary Figure S7). In particular, a significant increase in MMC-induced breaks, triradials, and quadriradials were observed in the Ku70^3A/3A^ MEFs compared to Ku70^+/+^ MEFs. Previously, we observed that mutating the putative human Ku70 phosphorylation sites did not result in a significant decrease in NHEJ (19). To assess if mutating the putative Ku70 phosphorylation sites in mouse Ku70 affects NHEJ, we examined IR-induced 53BP1 foci formation and resolution in G1 cells as an indirect assay for NHEJ. As shown in Figure 6F and Supplementary Figure S8, there is no difference in 53BP1 foci induction/resolution at 1 and 2 h post-IR in the Ku70^3A/3A^ MEFs compared to Ku70^+/+^ MEFs. However, a slight, but significant decrease in 53BP1 foci resolution is observed in Ku70^3A/3A^ MEFs at later time points (4 and 6 h post-IR), suggesting there is a modest decrease in NHEJ in the Ku70^3A/3A^ MEFs. Moreover, IR survival assays in the presence of the DNA-PK_cs_ inhibitor NU7441 showed that Ku70^3A/3A^ MEFs are more sensitive than Ku70^+/+^ MEFs, implicating that NHEJ is not fully defective in Ku70^3A/3A^ MEFs and that the increased radiosensitivity is due to decreased HR and NHEJ (Figure 6E). Collectively, the data presented in this manuscript illustrate that mutating the putative Ku70 phosphorylation sites in a mouse model results in decreased DNA repair capacity, which induces chronic DNA damage and genomic instability and this subsequently drives the induction of spontaneous HCC.

## DISCUSSION

Liver cancer is the fifth leading cause of cancer-related deaths (42). Among primary liver cancers, HCC is the most common subtype and is associated with multiple risk factors, including hepatitis B or C infection, alcohol consumption, gender (male), metabolic disorders (obesity and diabetes), and diet contamination (aflatoxins) (42). The major HCC risk factors damage DNA either directly, such as DNA alkylating agents, or indirectly via inflammation, which promotes the release of DNA damaging ROS. The promotion of DNA damage and genomic instability are closely associated with HCC development (43). However, the DNA repair factors required for protecting the liver from DNA damage and thus suppress HCC development and progression are mostly unknown.

Mouse models have allowed groups to study the physiological functions of DSB repair proteins and to characterize their roles in protecting against cancer (44). Studies using mouse models examining the role of the DDR machinery in modulating liver carcinogenesis are limited, but previous reports have implicated the Ku heterodimer in protecting against liver tumorigenesis. For example, deletion of either Ku protein does not result in increased overall cancer levels, but one mouse in the Ku70^−/−^ cohort developed HCC at 40 weeks (12). Furthermore, Ku70^−/−^ mice had accelerated DEN-induced HCC development compared to Ku70^+/+^ and Ku70^+/–^ littermates (21). Ku80^+/–^PARP^−/−^ mice develop liver cancers, including HCC (20). In this study, we further establish a role in Ku for protecting against HCC induction. Here, we developed a mouse model using C57BL/6J mice in which we blocked phosphorylation of the three conserved putative Ku70 phosphorylation sites by mutating them to alanine. The goal was to examine if blocking Ku70 phosphorylation attenuated HR, resulting in genomic instability and carcinogenesis. The Ku70^3A/3A^ mice are distinct from Ku70^−/−^ (null) mice, as ablating the putative Ku70 phosphorylation sites does not drive gross defects in immune cell production or premature aging, suggesting that V(D)J recombination and telomere maintenance are not affected, respectively (10–12). However, Ku70^3A/3A^ and Ku70^3A/+^ developed spontaneous HCC and Ku70^3A/3A^ mice had accelerated DEN-induced HCC compared to Ku70^+/+^ mice. Liver tumors are extremely rare in untreated C57BL/6J male mice; the incidence of liver tumors by 2 years of age is <5%, and DEN-induced HCC is also lower in C57BL/6J compared to other strains (35–37,45). Since the C57BL/6J mouse line is resistant to liver carcinogenesis, our data indicate that the Ku70 3A mutations specifically drives HCC development and is not due to intrinsic increased susceptibility of the mouse strain. Logistic regression analysis found spontaneous HCC tumor odds to be 33.6 times larger in Ku70^3A/3A^ compared to Ku70^+/+^ mice, showing the significance of the Ku70 3A mutations in promoting HCC. To our knowledge, this present study is also the first to report spontaneous induction of HCC due to point mutations in a DDR protein. Lastly, the data presented here shows that blocking putative Ku70 phosphorylation results in increased spontaneous and DEN-induced HCC, which together with the data in the literature, suggests that the Ku heterodimer plays a key function in suppressing HCC and strengthens its perceived role as a tumor suppressor.

The role of Ku in the pathogenesis and development of human HCC remains unclear. One report found that Ku80 is frequently downregulated in human HCC (46). However, a different study found that Ku70 upregulation correlated with HCC and was associated with poor prognosis (47). Differential expression of the canonical NHEJ factors, Ku70, Ku80, DNA-PK_cs_ and XRCC4, correlates with increased susceptibility to HCC (22). Sequencing of tumor samples has been beneficial in identifying mutations in DDR genes that are potential drivers of carcinogenesis, including HCC. Data mining of the COSMIC (Catalogue of Somatic Mutations in Cancer) and TCGA databases identified multiple single nucleotide variations in the *XRCC6* (Ku70) gene in and near the Ku70 phosphorylation cluster, including at the conserved phosphorylation sites S314 (endometrial cancer) and T316 (colorectal cancer) (Supplementary Figure S9). Mutations at G309 and S324 were identified in patients with HCC (Supplementary Figure S9). Collectively, this supports the notion that the Ku70 phosphorylation sites and amino acid near these sites are important for protecting against carcinogenesis. Unfortunately, many targeted sequencing programs do not include the canonical NHEJ factors in their ‘key cancer genes’, including the Memorial Sloan Kettering-Integrated Mutation Profiling of Actionable Cancer Targets (MSK-IMPACT) (48). As there is interplay between the DNA repair pathways and the fact that mounting evidence, including this study, suggests the DNA-PK complex plays a role in protecting against tumorigenesis and cancer progression, we recommend adding the canonical NHEJ factors, in particular *XRCC6* (Ku70), *XRCC5* (Ku80) and *PRKDC* (DNA-PK_cs_), to the sequencing studies examining mutations of DDR proteins in carcinogenesis (22,49).

We propose the following model for the role that Ku70 phosphorylation plays in protecting cells from HCC (See Graphical Abstract). The liver is constantly under attack from multiple agents, such as carcinogens (aflatoxins), metabolites (aldehydes from metabolism), and reactive oxygen species (ROS) from inflammation due to obesity. We postulate that these agents generate various DNA damage including DNA adducts, SSBs, DSBs, and ICLs. In Ku70^+/+^ mice, the DNA damage is rapidly repaired and genomic stability is maintained in the liver. However, in Ku70^3A/3A^ mice, mutating the putative Ku70 phosphorylation sites attenuates the repair of DSBs that are directly generated or those created due to the processing of DNA adducts, SSBs, or ICLs. This is supported by our data showing that the HCC tumors identified in Ku70^3A/3A^ mice have increased unrepaired DSBs as assessed by γH2AX staining and Ku70^3A/3A^ MEFs are sensitive to IR and MMC compared to MEFs from wild-type littermates. We postulate that the decrease in DSB repair capacity observed in Ku70^3A/3A^ mice and MEFs is due to a marked reduction in HR and a slight decrease in NHEJ. The decrease in HR is due to sustained retention of the Ku70 3A protein at DSBs, which attenuates DNA end resection. Recently, we published a study (50) that reports the small-angle X-ray scattering (SAXS) structure of the human Ku heterodimer with either Ku70 5A protein or wild-type Ku70. The scattering data shows the Ku70 5A protein has a profile skewed to the right consistent with a more extended/dynamic structure compared to the wild-type protein, which may explain the modest decrease in NHEJ observed in the Ku70^3A/3A^ MEFs. Ku70^3A/3A^ mice and MEFs show sensitivity to agents that generate multiple types of DNA damage including DEN (DNA adducts, DSBs, SSBs, and ICLs), IR (SSBs, DSBs, and base damage), and MMC (ICLs and ROS-induced DNA damage), which supports the notion that Ku70^3A/3A^ mice may have issues repairing multiple types of DNA damage (19). One of the main drivers of repair of MMC-induced DNA damage is the FA pathway. FA patients have a predisposition to liver tumors and *FANCD2^–^^/^^–^* mice develop hepatic adenoma and HCC, supporting a role that disrupting the FA pathway and thus the repair of ICLs promotes HCC induction (51–55). Acetaldehyde, an endogenous and alcohol-derived metabolite, generates DNA damage including DNA crosslinks and adducts that when processed can produce DSBs (56). In mice, Ku70 cooperates with the FA pathway in mediating the cellular resistance to endogenous and acetaldehyde-induced DNA damage (57). Ku70^3A/3A^ tumors have increased 8-oxo-G staining, which suggests that the putative Ku70 phosphorylation sites may also be important for the repair of base damage generated in the liver. In a p53-mutant background, Ku70^−/−^ mice exhibited an elevated level of point mutations and increased chromosomal rearrangements in liver, suggesting a defect in repair of base damage (58). The Ku heterodimer has 5′dRP/AP lyase activity and is essential for efficient removal of AP sites near DSB ends, and we speculate that mutating the putative phosphorylation sites on Ku70 may affect Ku's 5′dRP/AP lyase activity (59). Collectively, we postulate that mutating the putative Ku70 phosphorylation sites results in accumulated DNA damage, in particular DSBs, genome instability, spontaneous neoplasia, and finally HCC.

## DATA AVAILABILITY

All study materials will be made available to other researchers. Please contact Anthony J. Davis (anthony.davis@utsouthwestern.edu) for reagents.

## Supplementary Material

gkab743_Supplemental_File
