# Supplementary material for: Ablating putative Ku70 phosphorylation sites results in defective DNA damage repair and spontaneous induction of hepatocellular carcinoma
Source: Nucleic Acids Res. 2021 Aug 24;49(17):9836–50. doi: 10.1093/nar/gkab743 (PMC8464062; doi:10.1093/nar/gkab743)
Supplement: gkab743_Supplemental_File [file gkab743_supplemental_file.pdf]

Residue positions marked above the alignment: 305, 306, 307, 314, 316

H. sapien	296-VKTKTRTFNTSTGGLLLPSDTKRSQIYGSRQ-326-	human
P. troglodyes	296-VKTKTRTFNTSTGGLLLPSDTKRSQIYGSRQ-326-	chimpanzee
G. gorilla	296-VKTKTRTFNTSTGGLLLPSDTKRSQIYGSRQ-326-	gorilla
D. leucas	294-VKTKTRTFNVNTGSLLLPSDTKRSQTYGSRQ-324-	beluga whale
S. scrofa	292-VKTKTRTFNVNTGSLLLPSDTKRSQTYGNRQ-322-	pig
R. norvegicus	294-VKTKTRTFNVNTGSLLLPSDTRSLTGTRQIV-324-	rat
M. musculus	294-VKTKTRTFNVNTGSLLLPSDTKRSLTYGTRQ-324-	mouse
G. gallus	333-VKTKTRVFNGKTGSLLLPSDTKRAQTYGNRG-363-	chicken
V. komodoensis	294-VKTKTRTFSRETGGLLLPSDTKRAQIYGNRQ-324-	komodo dragon
O. tshawytscha	292-VRTKTRLYHTQTGSLLLPSDTKRVQVYAGRQ-322-	salmon
E. lucius	292-VRTKTRLYHTQTGSLLLPSDTKRAQVYASKQ-322-	northern pike
D. rerio	293-VRTKSRLFHTQTGGILLPNDTKRAQVYGQKQ-323-	zebrafish
X. laevis	294-VKTKTRIFHSNTGSLLLPSDTKRSQTYGNRQ-324-	horned frog
D. melanogaster	295-VRTKRVITVQDDGSQDIETGGWYTCNVGERD-335-	fruit fly
C. elegans	265-KIVKTSGYVKLEDSIRNRRDLKKSIEIGGEK-345-	worm
A. thaliana	307-VKVERS-YICTDTGAIMQDPIQRIQPYKNQN-336-	arabidopsis
S. cerevisiae	311-EAYSKRKFLNPITGEDVTGKTVKVVPYGDLD-341-	yeast

Supplementary Figure 1. Alignment of Ku70 DNA sequences across multiple organisms. Putative phosphorylation sites are in red.

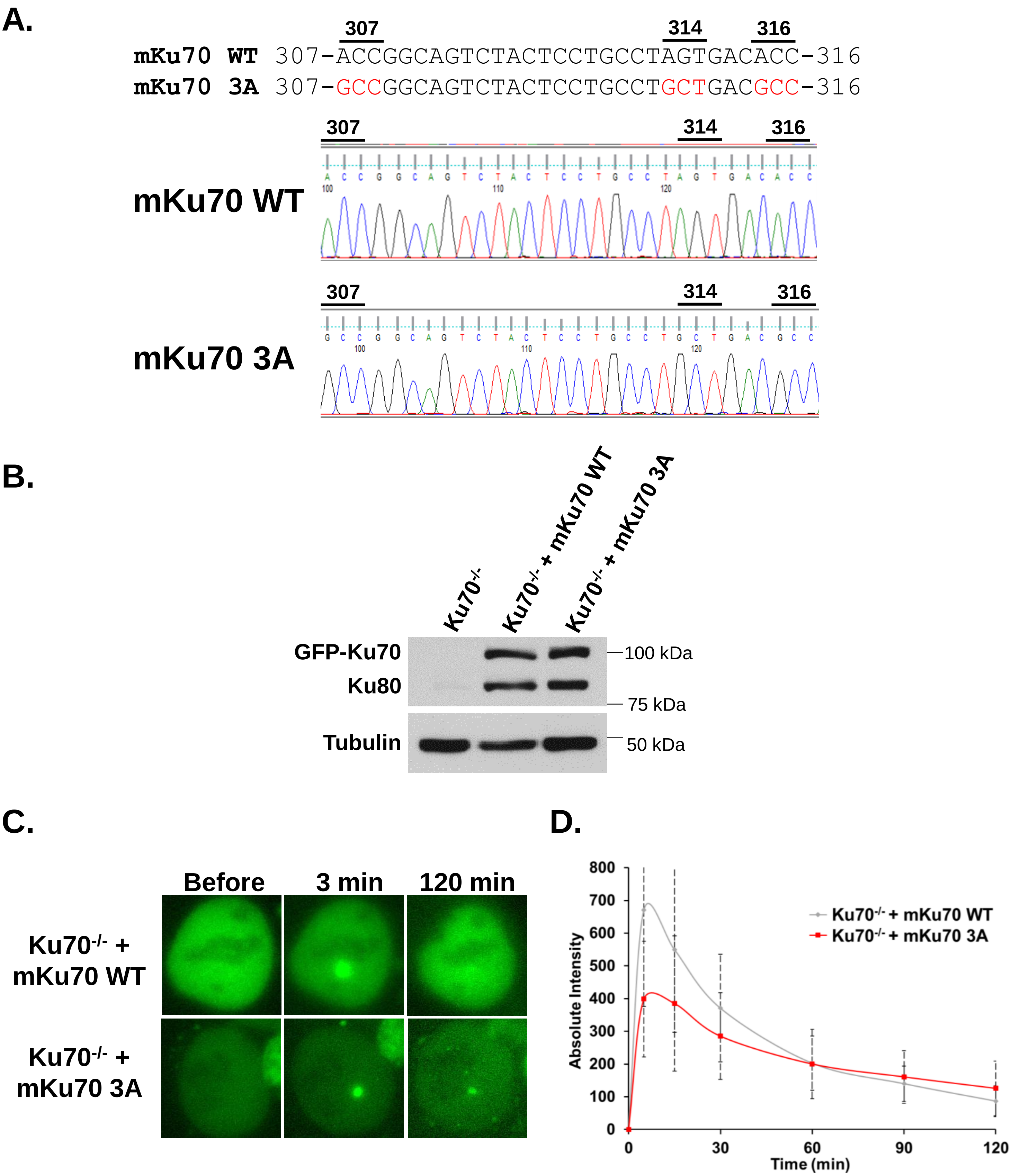

Supplementary Figure 2. A. Sequencing verification of site-directed mutagenesis of putative phosphorylation sites in mouse Ku70. **B.** Western blot analysis to verify the expression levels of Ku70 in DC-1 Ku70$^{-/-}$ MEFs complemented with either GFP-tagged mouse Ku70 wild-type (WT) or 3A. Ku70 and Ku80 were immunoblotted using Ku70 and Ku80 antibodies and anti-tubulin was used as a loading control. Representative images (**C**) and absolute intensity (**D**) of GFP-tagged Ku70 WT and 3A localization to laser-generated DSBs.

A.

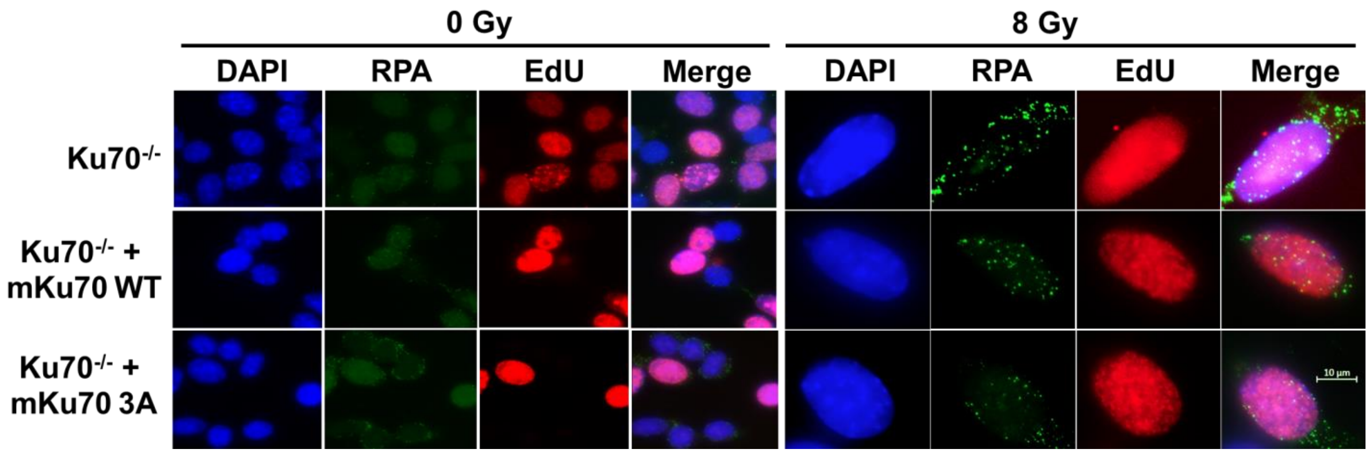

B.

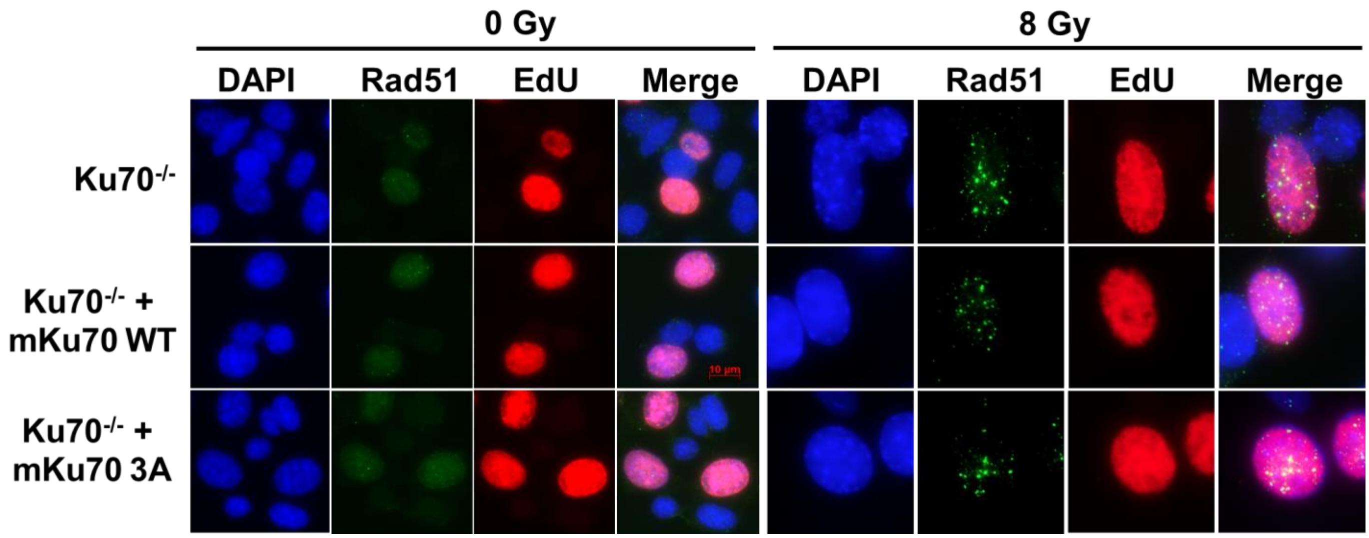

Supplementary Figure 3. Panel of images depicting RPA (**A**) and Rad51 (**B**) foci in Ku70$^{-/-}$ MEFs or Ku70$^{-/-}$ MEFs complemented with mouse Ku70 wild-type (mKu70 WT) or 3A (mKu70 3A) in EdU positive cells in untreated cells (0 Gy) or 8 h after exposure to 8 Gy of γ-rays.

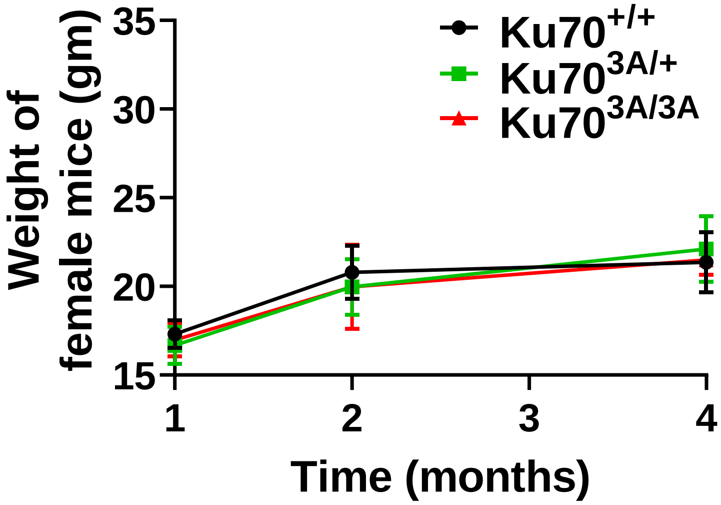

Supplementary Figure 4. Growth of female Ku70$^{+/+}$, Ku70$^{3A/+}$, Ku70$^{3A/3A}$ mice was tracked by weight. Error bars denote SD values for the weight of five mice of each genotype.

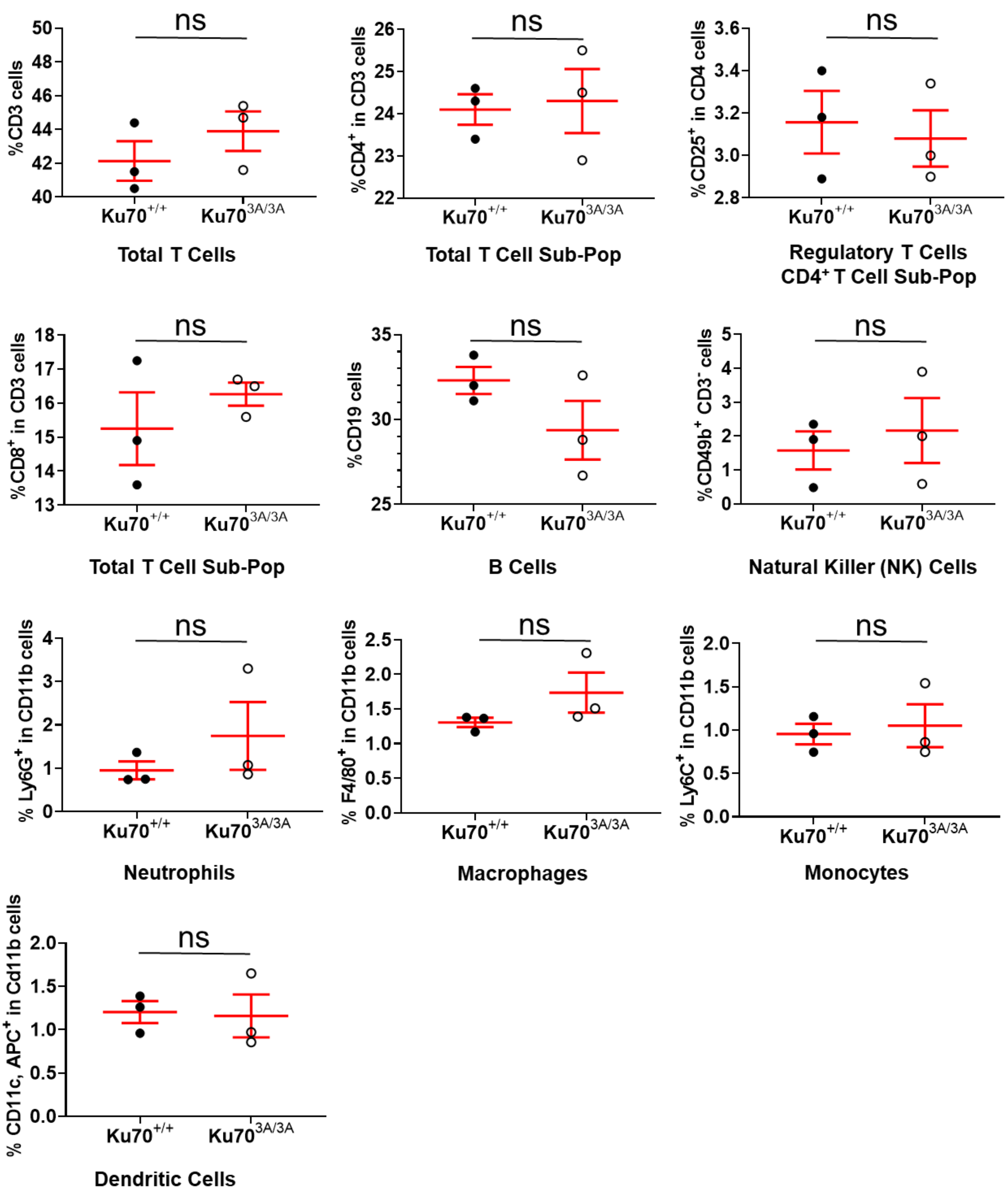

Supplementary Figure 5. Examination of splenic immune cells from 8-week old Ku70$^{+/+}$and Ku70$^{3A/3A}$ mice. Individual cell populations are marked in each panel. Three mice were used from each mouse genotype and Student t-test was used to examine statistical significance (ns = no statistical difference).

A.

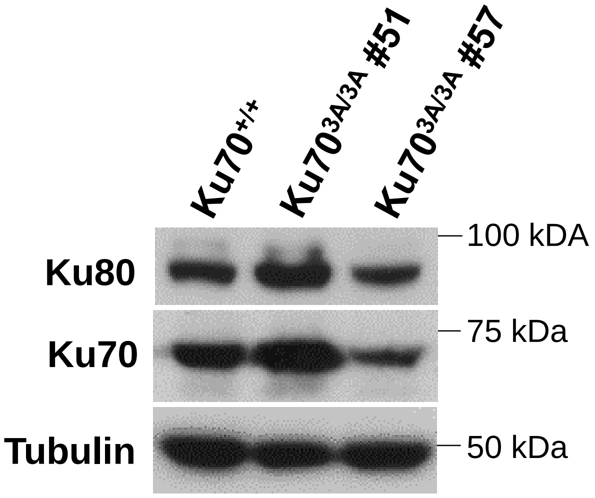

B.

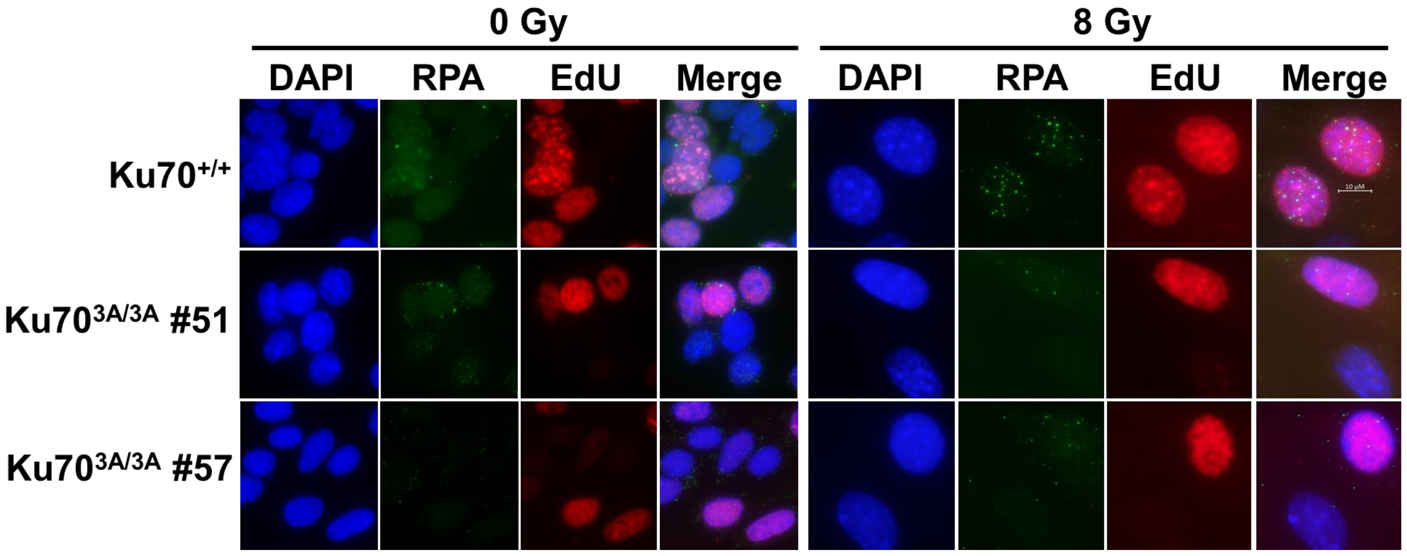

Supplementary Figure 6. A. Western blot analysis to examine the expression levels of Ku70 and Ku80 in MEFs isolated from Ku70$^{+/+}$ and two Ku70$^{3A/3A}$ mice. Ku70 and Ku80 were immunoblotted using Ku70 and Ku80 antibodies and anti-tubulin was used as a loading control. **B.** Panel of images depicting RPA foci in EdU positive cells in Ku70$^{+/+}$ and Ku70$^{3A/3A}$ MEFs in untreated cells (0 Gy) or 8 hours after treatment with 8 Gy.

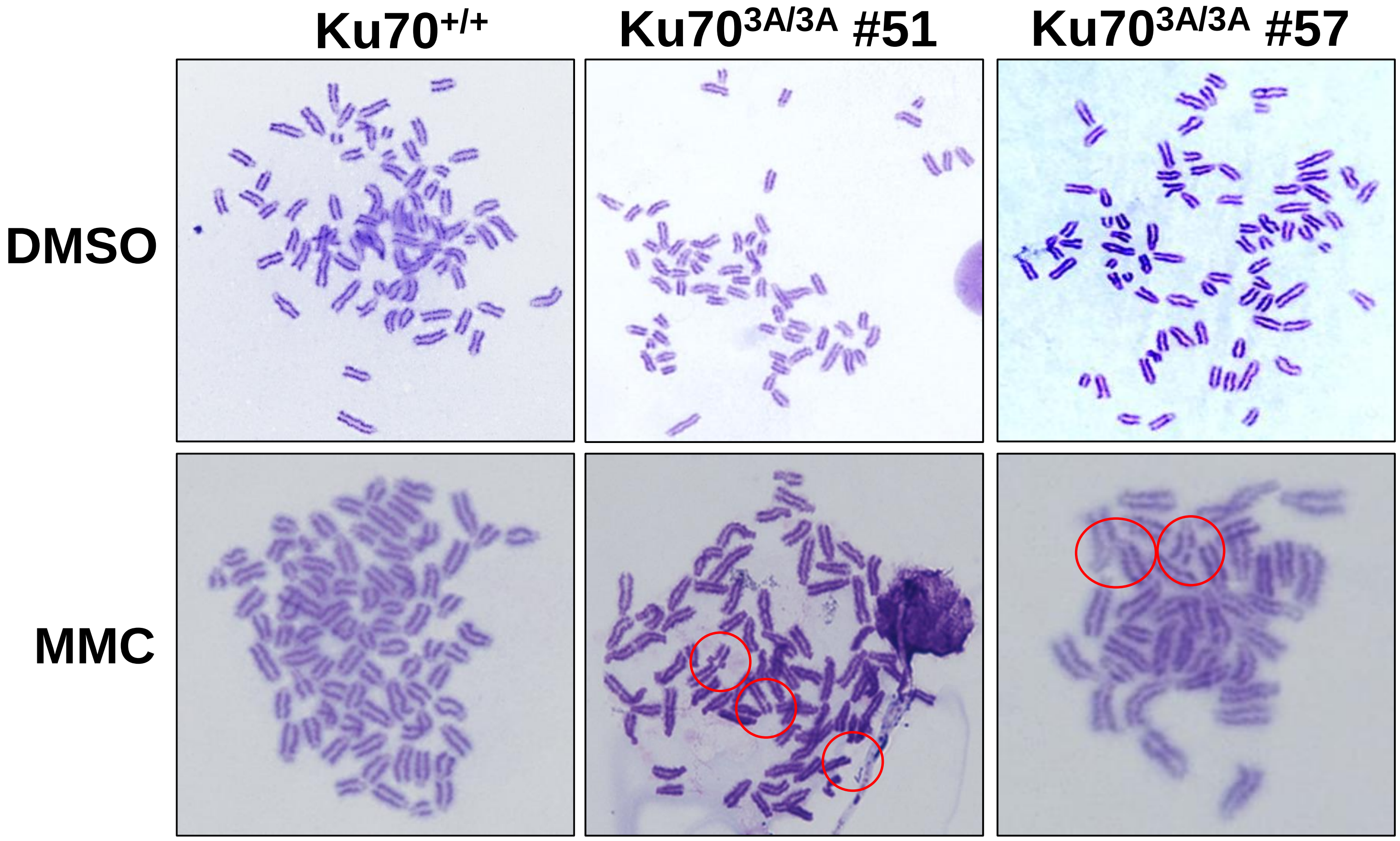

Supplementary Figure 7. Representative images of chromosomes following treatment with DMSO or MMC.

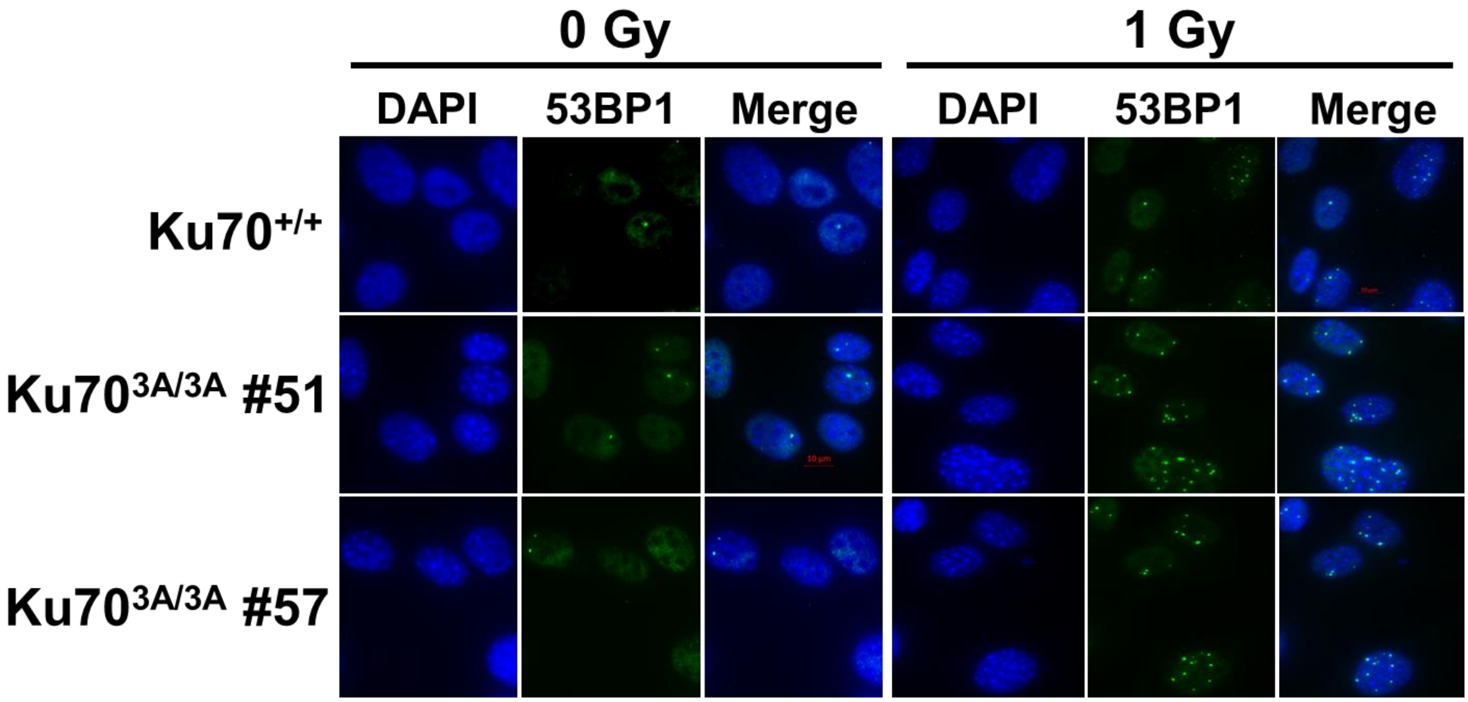

Supplementary Figure 8. Panel of images depicting 53BP1 foci in in $Ku70^{+/+}$ and $Ku70^{3A/3A}$ MEFs in untreated cells (0 Gy) or 8 hours following treatment with 1 Gy.

306
305 307 314 316

```
Human Ku70 296-VKTKTRTFNTSTGGLLLPSDTKRSQIYGSRQ-326
Mouse Ku70 294-VKTKTRTFNVNTGSLLLPSDTKRSLTYGTRQ-324
```

Mutation	Cancer Type
V296M	Melanoma
T302I	Ovarian
G309C	Liver
S314R	Endometrial
D315N	Glioblastoma
T316I	Colorectal
K317N	Myeloma
S324N	Liver
R325C	Colorectal

Supplementary Figure 9. Data mining of the COSMIC (Catalogue of Somatic Mutations in Cancer) and TCGA databases in order to identify single nucleotide variations in the *XRCC6* (Ku70) gene in and near the Ku70 phosphorylation cluster.
